# Genome-wide characterization and expression profiling of B3 superfamily during ethylene-induced flowering in pineapple (*Ananas comosus* L.)

**DOI:** 10.1186/s12864-021-07854-1

**Published:** 2021-07-21

**Authors:** Cheng Cheng Ruan, Zhe Chen, Fu Chu Hu, Wei Fan, Xiang He Wang, Li Jun Guo, Hong Yan Fan, Zhi Wen Luo, Zhi Li Zhang

**Affiliations:** 1 Key Laboratory of Tropical Fruit Tree Biology of Hainan Province, Institute of Tropical Fruit Trees, Hainan Academy of Agricultural Sciences, Haikou, 571100 China; 2 College of Resources and Environment, Yunnan Agricultural University, Kunming, 650201 China

**Keywords:** Pineapple, Flowering, Ethylene, B3

## Abstract

**Background:**

The B3 superfamily (B3s) represents a class of large plant-specific transcription factors, which play diverse roles in plant growth and development process including flowering induction. However, identification and functional surveys of B3 superfamily have not been reported in ethylene-induced pineapple flowering (*Ananas comosus*).

**Results:**

57 *B3* genes containing B3 domain were identified and phylogenetically classified into five subfamilies. Chromosomal localization analysis revealed that 54 of 57 *AcB3*s were located on 21 Linkage Groups (LG). Collinearity analysis demonstrated that the segmental duplication was the main event in the evolution of *B3* gene superfamily, and most of them were under purifying selection. The analysis of *cis*-element composition suggested that most of these genes may have function in response to abscisic acid, ethylene, MeJA, light, and abiotic stress. qRT-PCR analysis of 40 *AcB3s* containing ethylene responsive elements exhibited that the expression levels of 35 genes were up-regulated within 1 d after ethephon treatment and some were highly expressed in flower bud differentiation period in stem apex, such as *Aco012003*, *Aco019552* and *Aco014401*.

**Conclusion:**

This study provides a basic information of *AcB3s* and clues for involvement of some *AcB3s* in ethylene-induced flowering in pineapple.

**Supplementary Information:**

The online version contains supplementary material available at 10.1186/s12864-021-07854-1.

## Background

Flowering has a direct impact on the reproduction and yield of plants [1]. Many transcription factors (TFs) are involved in flowering process, including APETALA2 (AP2), basic helix-loop-helix (bHLH), basic region leucine zipper (bZIP), and minichromosome maintenance1, agamous, deficiens and serum response factor (MADS) et al. [2, 3]. Pineapple [*Ananas comosus* (Linn.) Merr] is one of the four tropical fruits, which is widely planted in more than 80 countries or regions around world [4]. In practice, there are mainly two ways to induce the pineapple flowering. One is natural flowering induction that needs short day-length and cold night temperatures to produce endogenous ethylene, and the other is artificial flowering induction that adopts chemicals such as ethephon or calcium carbide to release ethylene [5–7]. Due to the non-uniformity of flowering in natural environment, artificial flowering induction is often used in production to effectively improve flowering rate of pineapple [8]. At present, some genes related to ethylene or flowering in pineapple have been studied, but the underlying mechanisms of ethylene-induced pineapple flowering remain largely unclear.

Exclusively presented in plants, the B3-type DNA binding domain (DBD) is firstly identified in *VIVIPAROUS1* (*VP1*) in *Zea mays* and found in all members of B3 superfamily (*B3s*) [9, 10]. The number of *B3s* varies among plants, and there are 118, 91, 81, 108 members in *Arabidopsis thaliana*, *Oryza sativa*, *Zea mays*, *Glycine max*, respectively [11, 12]. Based on the structure and function of the proteins, they can be divided into five subgroups: *auxin response factor* (*ARF*), *Leafy Cotyledon 2* (*LEC2*)-*Abscisic Acid Insensitive* 3(*ABI3*)-*VAL* (*LAV*), *high-level expression of sugar inducible* (*HSI*), *related to ABI3/VP1* (*RAV*) and *reproductive meristem* (*REM*) [13, 14]. In *A. thaliana*, *ABI3* (one B3 domain), *HSI* (one B3 domain and zf-CW domain), *ARF* (one B3 domain, auxin response factor, and AUX/IAA domain), *RAV* (one B3 domain and AP2 domain), and *REM* (two B3 domains) are characterized by having different typical domains [15]. Each subgroup of *B3s* plays diverse roles in plant growth, development, and stress responses [16, 17]. For example, ARFs have the functions in the development of flowers and leaves, vascular tissue differentiation and root initiation [18, 19]. In *A. thaliana*, either *arf1* or *arf2* mutant exhibits delayed development, including flowering initiation and rosette leaf senescence [20], while the double mutant *arf7 arf19* influences root growth and leaf expansion [21]. The study of *LAV* is few, mainly focusing on the regulation of seed germination, embryo development, and stress response [22–25]. *AtABI3* induces lateral root primordia by auxin, and the root carrying loss-of-function *abi3* alleles is insensitive to auxin [26]. The *LEC2* induces the somatic embryo formation and promotes the accumulation of seed storage protein and oil bodies [27]. As a transcriptional repressor, *HSI* has functions in repressing the expression of seed maturation genes [28]. Recently, a study demonstrated that *HSI2* represses *AGL15* (relate to seed development) by the PRC2 component MSI1, contributing to seed maturation regulation [29]. Many studies indicated that the *RAV* is related to stress response and overexpression of cotton *RAV1* gene in *Arabidopsis* increases the sensitivity of salinity and drought stresses [30]. From another perspective, *TEM* (*TEMPRANILLO*) gene (a member of the RAV family) downregulates the expression of *FT* (*Flowering Locus T*), so as to repress flowering [31]. While *AtREM34* was the first identified REM member, *VRN1* (*VERNALIZATION1/REM5*), was the first being functionally characterized to be related to vernalization and promotion of flowering [3, 32]. Silencing both *REM34* and *REM35* in *Arabidopsis* influenced its female and male gametophyte development, indicating that *REMs* play roles in flowering [33]. Despite the fact that *B3s* plays critical roles in regulating flowering, the genome-wide analysis of this superfamily in pineapple has not been reported. What’s more, it is unclear whether *B3s* involve in ethylene response and flowering in pineapple. In this study, we identified 57 *B3s* in pineapple, and explored features of their structure and expression. The results provides a reference for further understanding of the physiological function and mechanism of *B3s* in the process of ethephon-induced pineapple flowering.

## Results

### Genome-wide identification and phylogenetic analysis of AcB3s

**Fig. 1:**
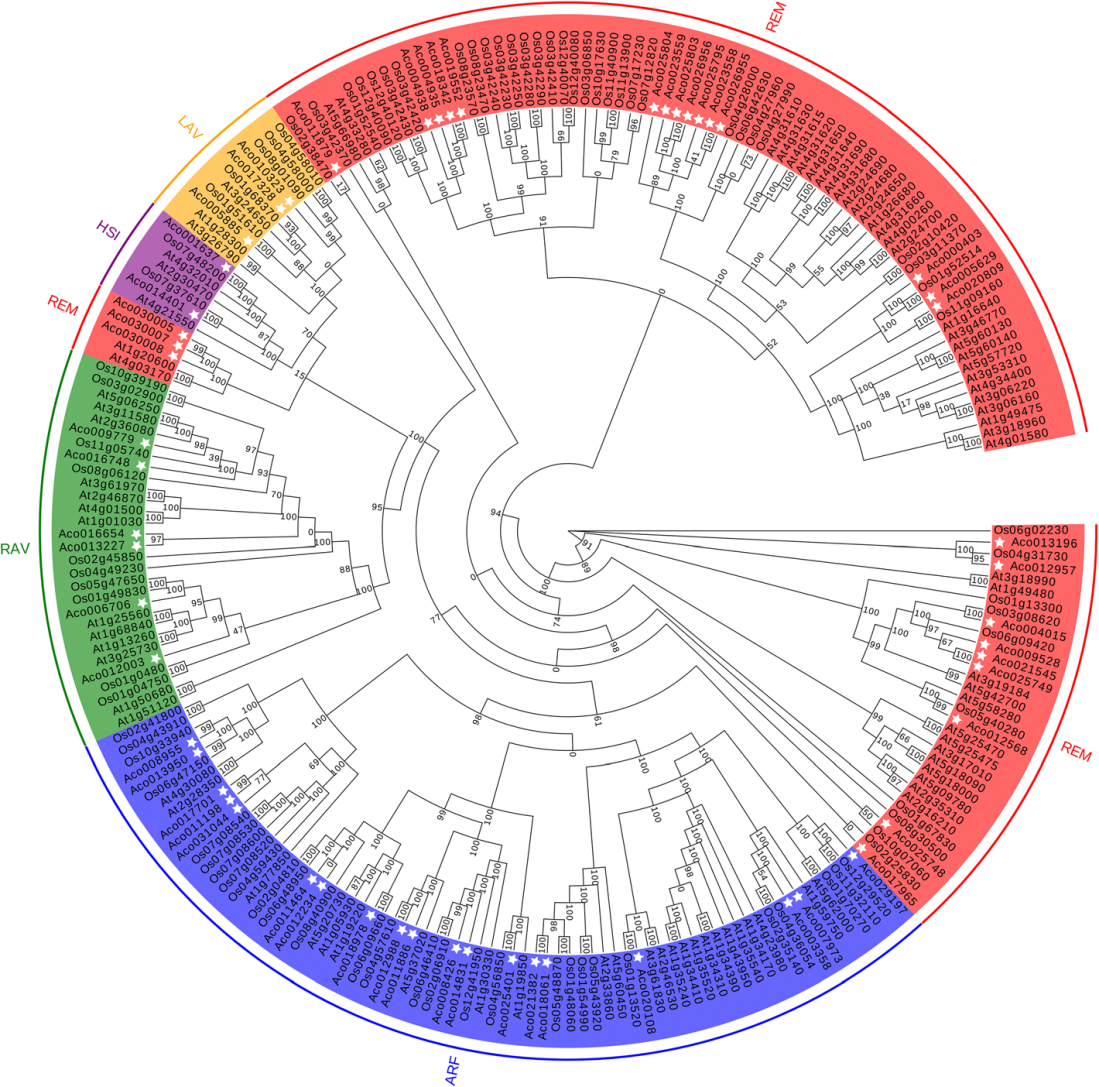
The phylogenetic tree of B3s from pineapple (Ac), *Arabidopsis* (At), and rice (Os). 87 in *Arabidopsis* [15], 86 in rice [15], and 57 in pineapple were utilized for the phylogenetic analysis. The different colour areas indicated different subfamlies. The white asterisk represented B3s from pineapple

A total of putative 57 B3 proteins were identified in pineapple by HMMER 3.0, CD-search and SMART analysis. As shown in Table S1, the number of amino acid residues of 57 B3 proteins ranged from 137 to 997 aa, and their molecular weights (MWs) varied from 15.71 to 111.32 KDa. All predicted B3 proteins were hydrophobic proteins with isoelectronic point (PI) values ranging from 4.61 to 9.9. The prediction of subcellular localization demonstrated that they were mainly located in nuclear (42), chloroplast (8), cytoplasmic (5), extracellular (1), and vacuole (1). A phylogenetic tree was constructed using PhyML 3.0 to investigate the evolutionary relationship of B3s among pineapple. The results indicated that 57 B3 proteins could be divided into five distinct subfamilies (REM, ARF, RAV, HSI and LAV) (Fig. 1). REM which clustered into three branches was the largest with 27 AcB3s, while ARF followed by REM with 19 AcB3s. Remainder subfamilies of RVA, HSI, and LAV had 6, 2, and 3 AcB3s, respectively.

### Genomic location and gene duplication analysis of AcB3s

**Fig. 2:**
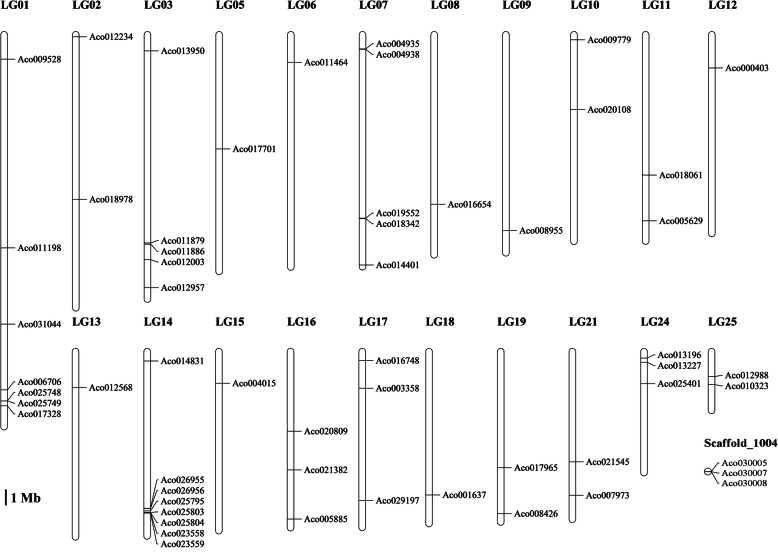
Distribution of *AcB3* genes on linkage groups. All 57 *B3* genes are mapped onto 21 linkage groups and one scaffold. “LG number” indicates the chromosome number. The scale is in megabases (Mb)

To examine the chromosomal distribution of the *AcB3s*, the gnomic sequence of each *AcB3* was utilized to search against the pineapple genome database with MapChart software (Fig. 2). 54 *AcB3s* were distributed on 21 out of the 25 Linkage Groups (LG), and the rest (Aco030005, Aco030007 and Aco030008) were located on scaffold_1004. However, the distribution of *AcB3s* in each chromosomal was uneven. LG14 contained 8 *AcB3s* (7 of them highly concentrated on 9.94 Mb–10.24 Mb of LG14), followed by 7 on LG01. Only one gene was observed on LG5, 6, 8, 9, 12, 13, 15 and 18.

**Fig. 3:**
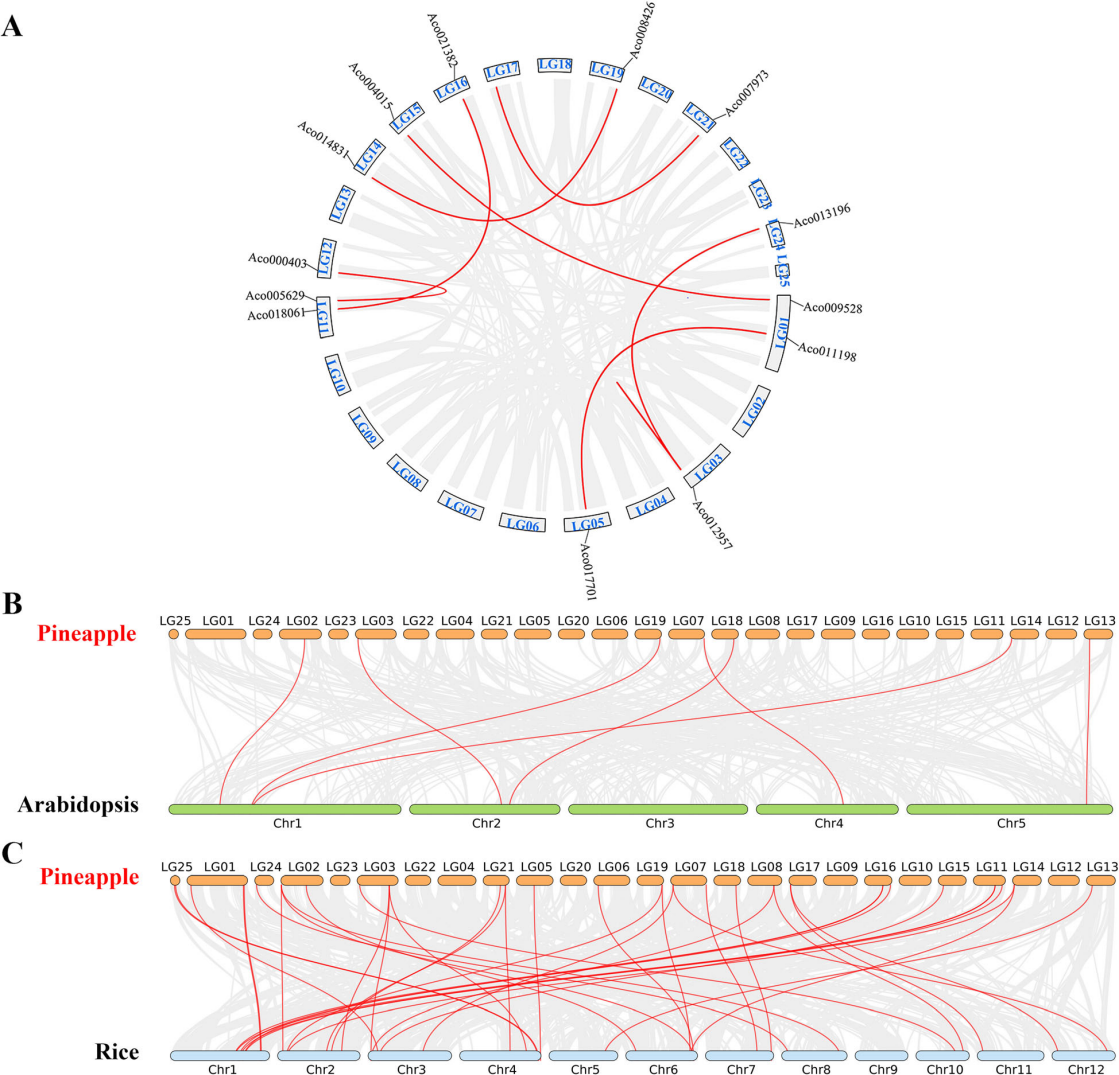
Collinearity analysis of *AcB3s*. A Collinearity analysis of AcB3s in pineapple. B Collinearity analysis between pineapple and *Arabidopsis.* C Collinearity analysis between pineapple and rice. Gray lines suggest all segmental duplications and the red lines suggest duplicated *AcB3* pairs. The chromosome number and gene ID are illustrated

Both BLASTP and MCScanX methods were used to identify the collinearity and potential duplication events of the *AcB3s* in pineapple. A total of 8 segmental duplication events were identified, and the synteny blocks of *AcB3* were on 12 LGs, including LG1, 3, 5, 11, 12, 14, 15, 16, 17, 19, 21 and 24 (Fig. 3A). Additionally, 5 tandem duplication events were identified in LG14 and scaffold_1044 (Table S2). Calculating the nonsynonymous (Ka) and synonymous (Ks) substitution rates is useful for the study of evolutionary. Among 13 duplication *AcB3* pairs, 2 of them were “Ka/Ks > 1”, suggesting that those had evolved under the effect of positive selection; 10 of them were “Ka/Ks < 1”, demonstrating that those had evolved under the effect of purifying selection, and the only one pair had Ks value equal to 0, which imply that they may be the redundant gene (Table S2). To further explore the potential evolutionary mechanisms of the *AcB3s*, we compared the collinear relationships between the pineapple and the other two species *Arabidopsis* (dicotyledon) and rice (monocotyledon) (Fig. 3B and C). 7 collinear gene pairs between pineapple and *Arabidopsis* and 39 collinear gene pairs between pineapple and rice were identified. The number of orthologous events of *AcB3s*-*OsB3s* was far more than that of *AcB3s*-*AtB3s*, indicating that the genetic relationship between pineapple and rice is closer compared to *Arabidopsis*. The details were illustrated in Table S3.

### Gene structural and protiein motif analysis of AcB3s

**Fig. 4:**
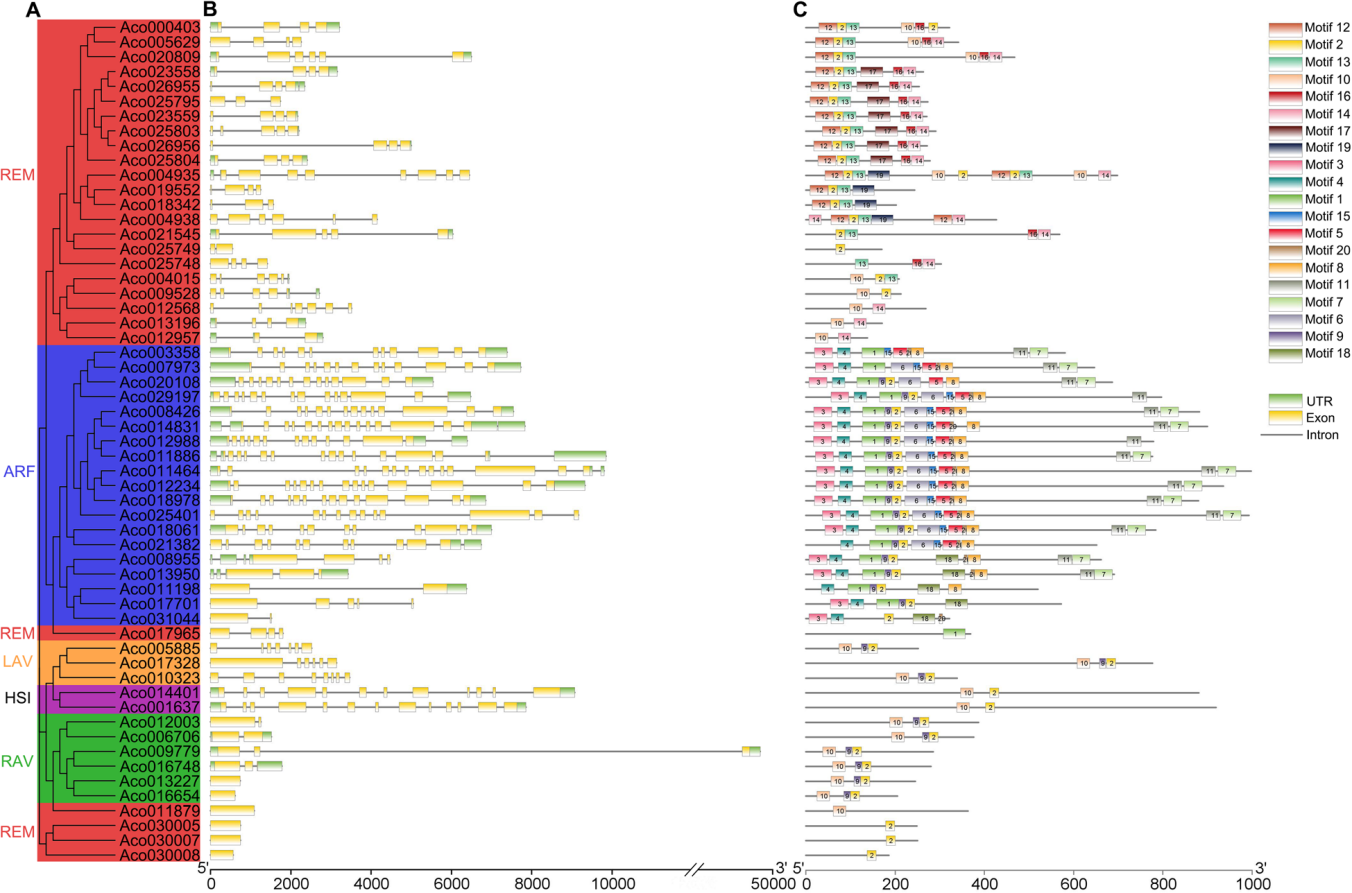
The exon-intron structure and motif organization of *AcB3s*. A Phylogenetic relationships of *AcB3s*. B The intron and exon structure of *B3* genes. The green and yellow boxes represent exons and UTRs, respectively, and the lines going through the boxes represent introns. C The motif identification of AcB3 proteins using MEME

Gene structure analysis is helpful to understand the functional evolution of genes in adapting to environmental changes [34]. Among 57 *AcB3s* divided into 5 subfamilies (Fig. 4A), most ARF subfamily genes had more than 11 exons, and more than half REM subfamily genes only had 3–5 exons. The LAV subfamily genes had 6–8 exons, the HSI subfamily genes had 12 or 13 exons, and the RAV subfamily genes had 1 or 2 exons (Fig. 4B). Otherwise the same subfamily also has the similar domains (Fig. S1). Additionally, motif analysis identified 20 divergent motifs in the protein sequences of *AcB3s* (Fig. 4C; Table S4). As demonstrated in the result, all *AcB3* genes had motifs annotated as B3 domain. The same group had similar motifs while motifs were diverse among different groups. For example, both *HSI* genes had motif 2 and 10 while all *ARF* genes had motif 3 and 4. Meanwhile, the motif 12, 13, 14 16, 17 and motif 19 only existed in the *REM* (Fig. 4C). The special motifs in groups may imply diverse functions of AcB3 superfamily.

### *Cis*-acting elements and gene ontology analysis of *AcB3s*

**Fig. 5:**
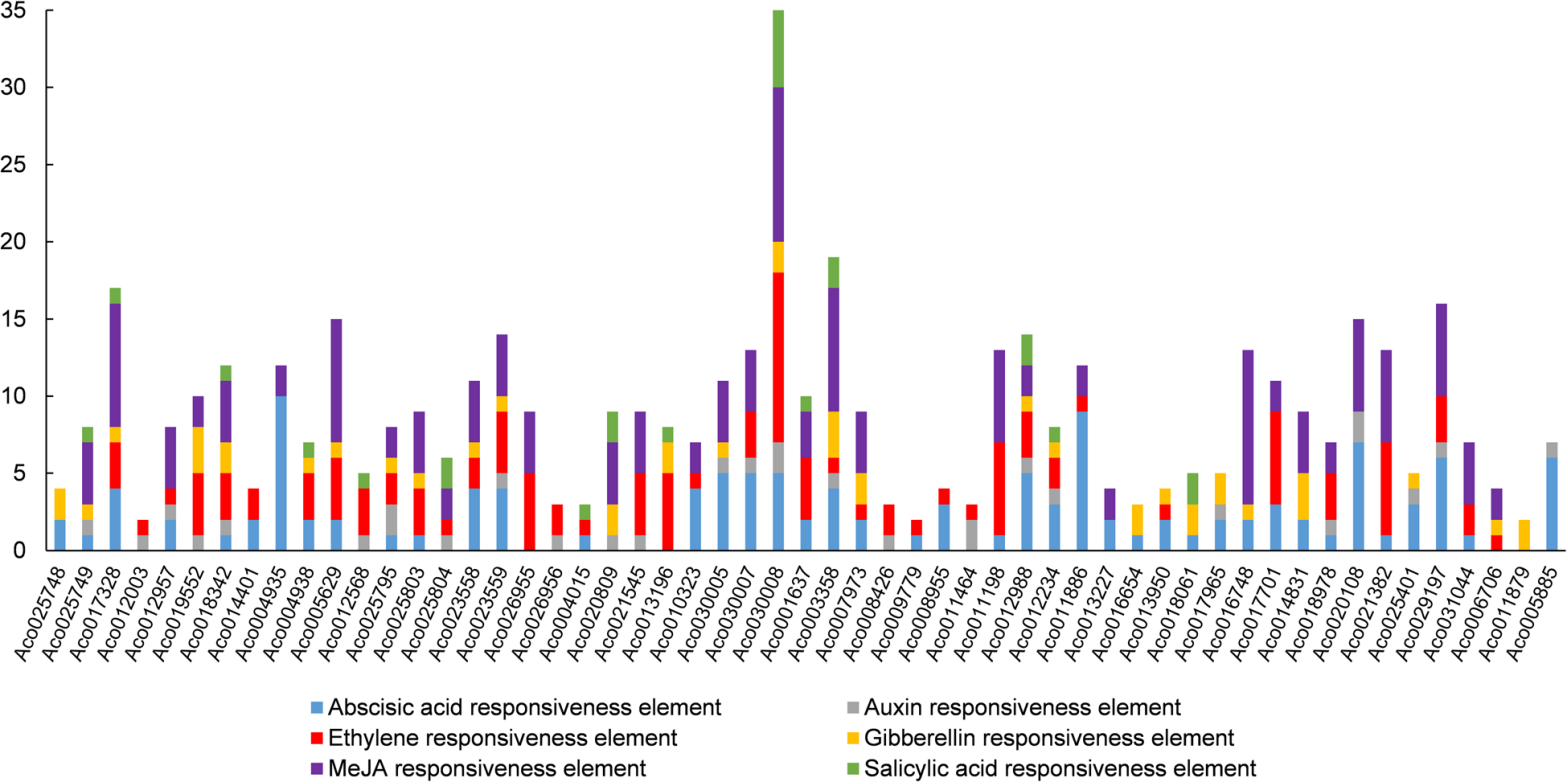
The prediction of hormone-related *cis*-elements in *AcB3* promoters. The promoter is 2.0 kb in length

In order to understand the possible regulatory roles of plant hormones in *AcB3s*, the 2000 bp-length promoter sequences before ATG were analyzed. As shown in Fig. 5, there were 42, 26, 40, 28, 35 and 15 *AcB3* promoter sequences with *cis*-elements related to abscisic acid, auxin, ethylene, gibberellin, methyl jasmonate and salicylic acid, respectively. Both *Aco009528* and *Aco000403* did not contain plant hormone-related *cis*-elements. Other *cis*-elements such as light responsiveness element, defense, and stress responsiveness element were also found (Table S5). Moreover, all *AcB3* promoter sequences had ERF binding sites (Table S6). We also executed a gene ontology (GO) enrichment analysis of the *AcB3s* (Fig. S2). The prediction of cellular component suggested that *AcB3s* participated in cell, organelle and protein-containing complex. The prediction of the biological process indicated that most *AcB3s* participated in DNA binding, and then transcription regulator or catalytic activity. Moreover, the prediction of molecular function demonstrated that *AcB3s* were mainly involved in metabolic process, cellular process, regulation of the biological process, and biological regulation. Additionally, two genes (Aco13950 and Aco006706) were predicted to function in the process of flowering in the terms on level 3 (Fig. S3; Table S7).

### Expression analysis of *AcB3* genes after ethephon treatment

**Fig. 6:**
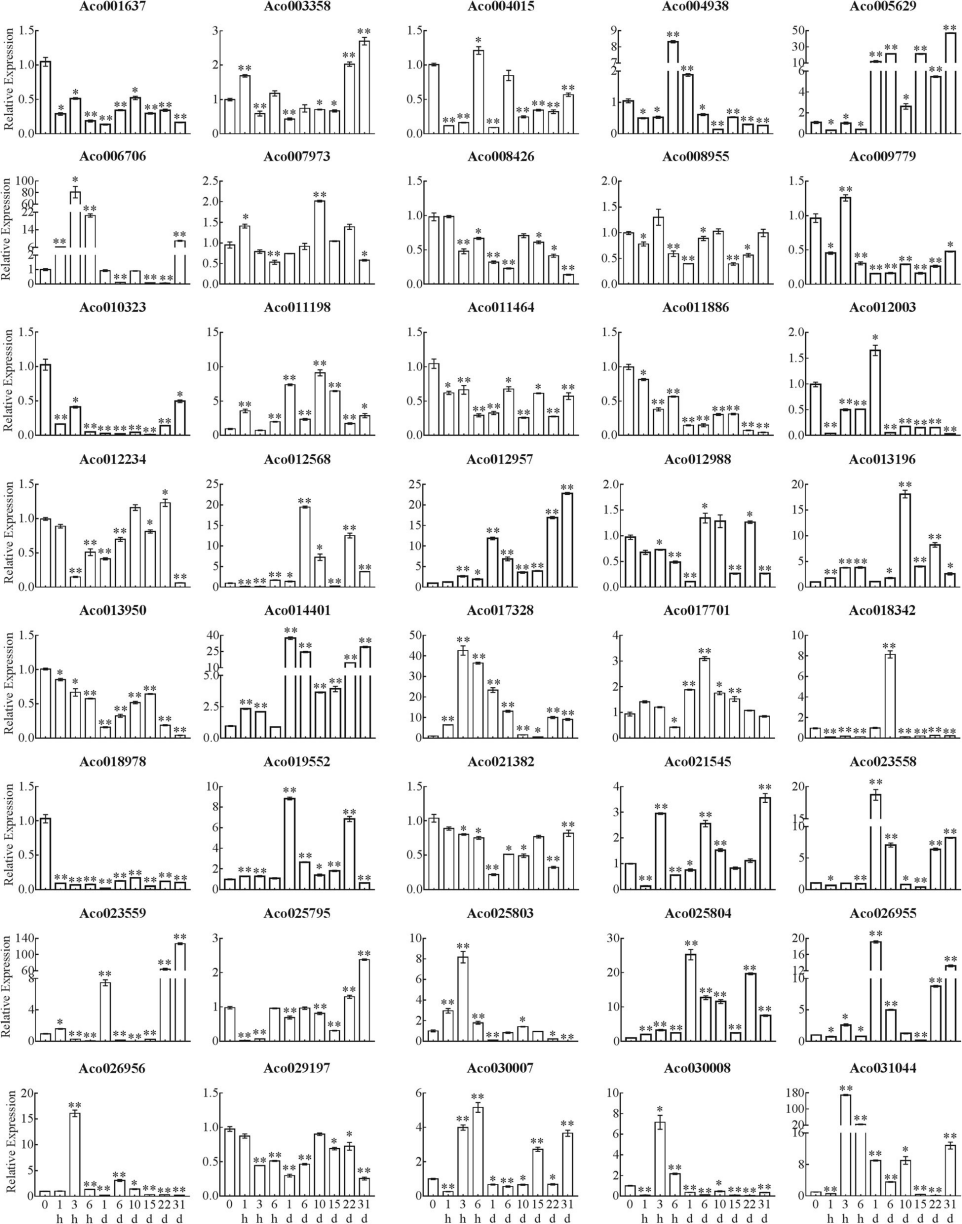
Expression profile of *AcB3*s in leaves after ethephon treatment. The *AcEF1* gene was used as an internal control. Error bars indicated standard error (SE) based on three replicates. *P < 0.05; **P < 0.01, student’s *t* test

**Fig. 7:**
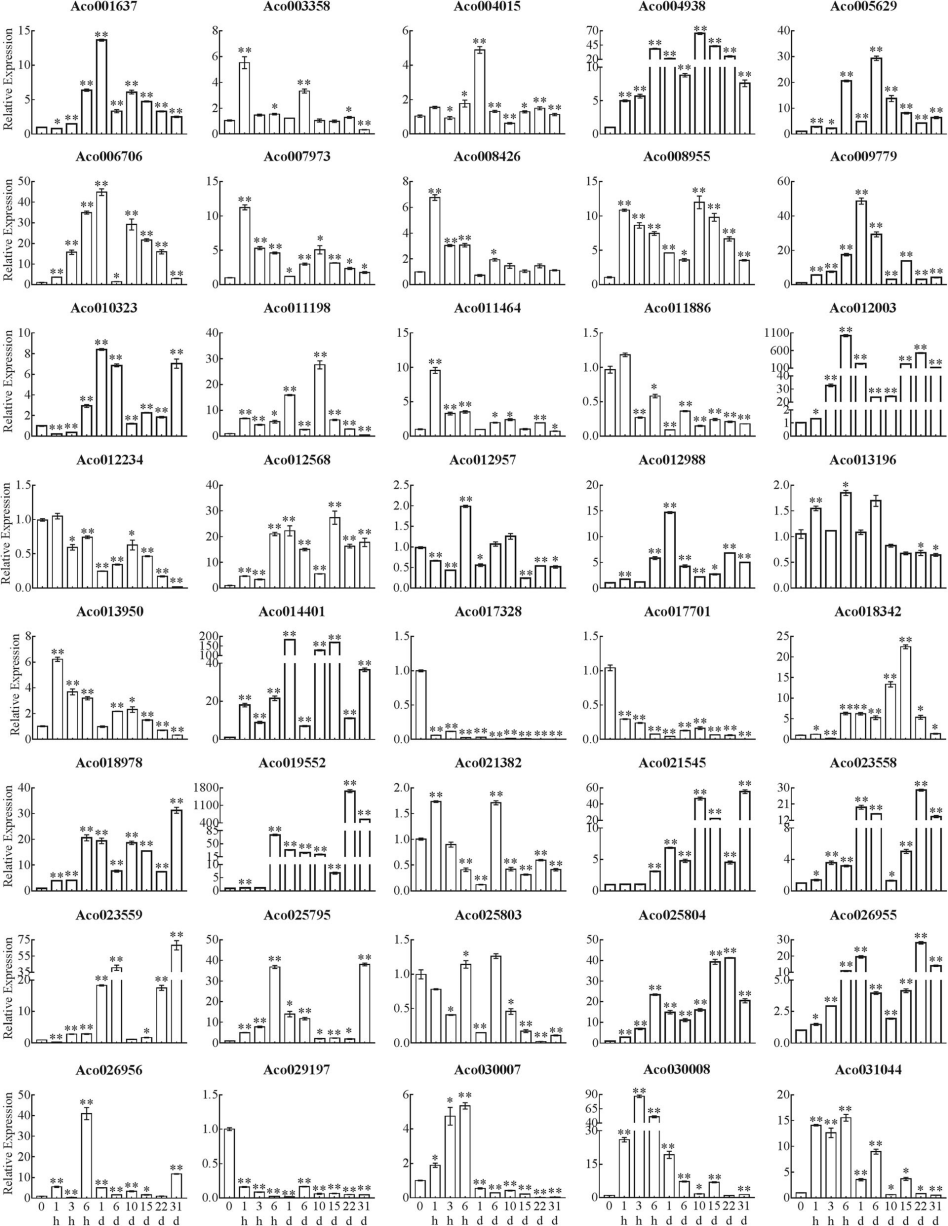
Expression profile of *AcB3s* in stem apex after ethephon treatment. The *AcEF1* gene was used as an internal control. Error bars indicated standard error (SE) based on three replicates. *P < 0.05; **P < 0.01, student’s *t* test

A previous study showed that flower buds of pineapple began to differentiate after 31 d of ethephon treatment [35]. In order to explore the dynamic expression pattern of *AcB3s* in response to ethylene within 31 d after ethylene treatment, 40 *AcB3s* covering all subfamilies and containing ethylene responsive elements were screened to analyze their expression changes by qRT-PCR. Generally, the expression of all the selected genes were significantly changed in response to ethephon. In leaves, the expression levels of 32 genes were significantly up-regulated while the expression levels of 8 genes were significantly down-regulated during the whole processing time. Especially, the expression levels of 16 genes (such as *Aco012957*, *Aco014401*, *Aco005629*, *Aco025804*, *Aco023558*, *Aco026955* and so on) were significantly up-regulated within 1 d and were highly expressed in 31d after ethephon treatment (Fig. 6). Compared with leaves, ethephon treatment induced up-regulated expression of more genes in stem apex. The expression of 35 of 40 genes were up-regulated from 0 to 1 d after ethephon treatment, of which 7, 6 and 7 genes expression reached a peak at 1 h, 6 h and 1d, respectively (Fig. 7). 25 genes in stem apex were significantly up-regulated in 31d after ethephon treatment. Particularly, the transcripts of 14 genes (Aco01637, Aco004938, Aco005629, Aco007973, Aco008955, Aco009779, Aco012003, Aco012568, Aco014401, Aco018978, Aco023558, Aco025795, Aco025804 and Aco026955) in stem apex were at a high level all the time, while the expression level of Aco019552 was the highest in stem apex.

## Discussion

*B3s* have various functions in regulating the development of root, seed and flower, abiotic stresses responses and involving in hormone signaling pathways [9, 14]. Genome-wide characterization and expression analysis of *B3s* were investigated in some plants, and discovered a lot of meaningful information [10, 17, 36, 37]. In this study, a total of 57 *AcB3s* were identified by a comprehensive genome-wide analysis. Although having the same five subfamilies, the number of *B3s* in pineapple were less than *Arabidopsis* and rice, demonstrating that the parallel evolutionary events of *B3*s also existed in the plants of Crassulaceae family. All 57 identified *AcB3s* had the typical B3 domains characteristic of *B3* genes. Consistent with the phylogenetic analysis, most genes had similar classification and number of motifs in the same subfamily, supporting the reliability of our subfamily classification.

57 *AcB3* genes were classified into five subfamilies by phylogenetic analysis. Like other species [11], the REM subfamily in pineapple also had the most genes followed by ARF subfamily (Fig. 1). It was reported that there are 20 *ARFs* in pineapple [38]. However, only 19 *ARF* genes were identified in our study, and *Aco015073* did not have the typical B3 domain although it had sequence similarity to one of the *ARFs*. Of the 19 *AcARFs*, 14 genes contained the B3, Aux_resp, and Aux_IAA domains, while the remaining genes had no Aux_IAA domain, indicating that conserved evolution in both B3 and Aux_resp domains. In addition, *Aco009779*, *Aco016748*, *Aco013227*, and *Aco016654* without AP2 domain were also classified as RAV subfamily. The RAV subfamily usually have one AP2 and one B3 domains, but not all RAV proteins contain AP2 domains [15, 39]. The number of B3 domains of REM subfamily in pineapple varies from 1 to 4, which may be caused by domain duplication events.

Tandem duplication, segmental duplication, and transposition are considered as the main gene duplication events promoting the expansion of gene families, among which segmental duplication contributes more than tandem duplication in plants [40, 41]. In this study, 8 segmental duplication events and 5 tandem duplication events were identified in *AcB3s*. Among them, the Ka/Ks value of 10 genes was less than 1 while the value of two genes was more than 1 (Table S2), demonstrating that positive selection happened in *AcB3s*.

As illustrated by previous studies, *AcFT* was highly expressed within 1 d and in flower bud differentiation period in stem apex after ethephon treatment, and overexpression of *AcFT* gene resulted in early flowering [37, 42]. In this study, 35 of 40 *AcB3s* were observed to be responsive to ethylene in stem apex within 1d after ethephon treatment, and 25 exhibited the significant up-regulation in 31d after ethephon treatment (Fig. 7), implying that these *AcB3*s may play roles in the process of ethephon-induced pineapple flowering. Most of REM subfamily genes are targets of key flowing transcription factors, which are preferentially expressed in reproductive meristems, such as *AP1*, *AP3*, *PI*, *LEAFY* and *SVP* [3, 43, 44]. In *Arabidopsis*, *AtREM22* is related to the stamen development while *AtREM5* (*VRN1*) is involved in flowering time control [45, 46]. In this study, it was revealed that the expression levels of most *AcREMs* in stem apex were higher than in leaves after ethephon treatment. Among them, the *AcREM* genes (*Aco005629* and *Aco020809*) had the close genetic distance and similar structure with *AtREM17* (At4g34400), a target of *LEAFY* expressed preferentially in flowers [3, 10]. Thus, it is speculated that they may have the similar functions in the flower development of pineapple. *AtVAL1* (At2g30470) also named as *AtHSI2* is a target of *FLC* (*Flowering Locus C*) in the flowering process [47]. *Aco001637* having closely clusters with *At2g30470* was largely up-regulated in stem apex after 6 h ethephon treatment and reached to the higher level when treated for 31d (Fig. 1; Fig. 7), indicating that Aco001637 may function in the process of ethephon-induced flowering in pineapple. Auxin response mediated by *ARF* genes participates in flowering process [13]. In tomato, most *ARFs* had the higher expression levels in flower buds and flowers [48]. *Arabidopsis ARF6* and *ARF8* (At5g37020) coordinate the transition from immature to mature fertile flowers [49]. Interestingly, we found that ethylene up-regulated *Aco012988* was classified together with At5g37020. In addition, phosphorus is necessary for plant growth and development, and the concentration of phosphorus has an influence on flowering [50]. *OsARF16* (Os06g09660) in rice involves in phosphate transport [51]. Intriguingly, *Aco018978* was classified together with *OsARF16* in rice, and up-regulated significantly in 31d after ethephon treatment. It is reasonably to speculate that *Aco018978* in pineapple may participate in the regulation of flowering by increasing the phosphate transport. The variety used in the published genome was F-153 which belonged to Cayenne, and this study we used ‘Tainong 16’ which comes from Smooth Cayenne (Cayenne) × Rough (Queen) [52, 53]. Based on the genome sequences, we preliminary studied the expression of *AcB3* genes in ‘Tainong 16’, in order to demonstrate the expression divergence with other varieties, more experiments need to be carried out next.

## Conclusions

In this study, 57 *AcB3* genes were identified in the pineapple genome, and classified into five major classes (*REM*, *ARF*, *RAV*, *HSI* and *LAV*). Uneven distribution and segmental duplications on chromosomes contributed to the expansion of *AcB3s*. Several candidate genes that contain ethylene response elements and are significantly up-regulated by ethylene in stem apex were identified. These results will provide a reference for future research on the molecular mechanisms of ethylene-induced pineapple flowering.

## Methods

### Identification and analysis of B3s in pineapple

To identify *AcB3s*, the genome of *Ananas comosus* were downloaded from the PGD (Pineapple Genomics database) (http://pineapple.angiosperms.org/pineapple/html/index.html) [54]. The HMM (hidden Markov model) matrix of B3 domain (PF02362) was downloaded from Pfam, and then *B3s* were retrieved by Hmmer 3.0 software. These predicted B3s proteins were further confirmed and analyzed using the CD-search (https://www.ncbi.nlm.nih.gov/Structure/cdd/wrpsb.cgi) and SMART (http://smart.embl-heidelberg.de) web server. All pineapple *B3s* contained B3 domain.

### Phylogenetic analysis

Multiple sequence alignments and phylogenetic tree of full-length B3s proteins from pineapple, *Arabidopsis* and rice were performed using PhyML 3.0 with maximum likelihood (ML) method, ‘AIC’ criterion and ‘aLRT Chi2-based’ as fast likelihood-based method [55]. The classifications of AcB3s were given according to the lists of AtB3s and OsB3s came from previous study [15]. The sequences of AtB3s and OsB3s were downloaded from Phytozome 12.1.6 (https://phytozome.jgi.doe.gov/pz/portal.html).

### Chromosomal locations and synteny analysis

The physical locations of *AcB3s* were obtained from the PGD. The location images of *AcB3s* were drawn by MapChart software [56]. Multiple Collinearity Scan toolkit (MCscanX) was employed to analyze the tandem and segmental duplications of *B3s* [57]. The diagrams of synteny analysis were drawn by TBtools [58]. The value of Ks and Ka were calculated by DnaSP 5 software [59].

### Structural and motif analysis of AcB3s

The exon/intron structures of the *AcB3s* were drawn by TBtools. The conserved motifs were analyzed by MEME (http://meme-suite.org/tools/meme) [60], searching up to 20 conserved motifs and each motif was set from 6 to 50 residues. Protein domains were identified using the NCBI CD-search and SMART.

### Analysis of *cis*-acting elements and gene ontology

The 2 kb promoter region upstream of the start codon of each gene was downloaded from PGD to analyze the possible *cis*-acting elements of *B3s* by Plant CARE (http://bioinformatics.psb.ugent.be/webtools/plantcare/html/) online server. The ERF binding sites were predicted by JASPAR (http://jaspar.genereg.net/), and the relative profile score threshold was ‘80’. WEGO (http://wego.genomics.org.cn/) was used to analyze Gene ontology of B3 proteins.

### Plant materials and treatments

The pineapple hybrid variety was “Tainong 16”. The temperature, humidity and other environmental conditions in the greenhouse which controlled in the appropriate range for pineapple flowering. The pineapple which grew 25 mature leaves (more than 30 cm in length) were treated by 80 mL of 1600 mg/L ethephon solution, and three biological replicates were sampled at 0, 1 h, 3 h, 6 h, 1 d, 6 d, 10 d, 15d, 22 d and 31 days, including leaves and stem apex. The leaves (medial green part) and stem apex were collected from three individual plants as a replication. The sample treated with water of the same volume as the contrast (0), all of them were frozen in liquid nitrogen and stored in refrigerator at − 80 °C until used.

### RNA isolation and *AcB3s* expression analysis

Total RNA was extracted from samples using Total RNA was extracted from samples using a universal plants RNA extraction kit for HuaYueyang, according to the manufacturer’s guidelines. 1 μg RNA were used to synthesize the first-strand cDNA by MonScript™ RTIII All-in-One Mix with dsDNase. Primers were designed for real-time quantitative PCR (qRT-PCR) using Primer 5.0 software, and the primer sequences were shown in detail in Table S8. The cDNA was used as the template, the results [ΔΔCT = (Ct_target gene_-Ct_*AcEF1*_) - (Ct_0_-Ct_*AcEF1*_)] were normalized against the Ct value of *AcEF1* based on three biological replicates. The reaction was carried out on Roche Lightcyler® 480 instrument using SYBR Green Master Mix (Roche). The reaction system of real-time quantitative PCR was 10 μL, including 1 μL of cDNA, 5 μL of SYBR Green I master, 0.5 μL of 10 μmol/L of forward and reverse primers, and 3 μL of ddH_2_O. The relative expression data were analyzed by 2^-ΔΔ CT^ method [61], and by Excel and Graphpad prism 7.0 software. The *t* test was analysed by SPSS 21 software.

## Supplementary Information

**Additional file 1: Fig. S1.** Structural domain diagram of B3 genes. **Fig. S2** Gene ontology analysis of AcB3 proteins. Three categories (cellular component, biological process and molecular function) and terms on level 2 were exhibited by different colors. **Fig. S3.** GO terms on level 3.

**Additional file 2: Table S1.** Physical and chemical properties of B3 genes predictions. **Table S2.** Ka/Ks calculation of the duplicated pineapple B3 gene pairs. **Table S3.** One-to-one orthologous relationships between pineapple and *Arabidopsis*, pineapple and rice. **Table S3.** One-to-one orthologous relationships between pineapple and *Arabidopsis*, pineapple and rice. **Table S4.** Seauence and characteristics of conserved motifs identified in pineapple. **Table S5.** The *cis*-element sites of 57 *B3* promoters. **Table S6.** The prediction of ERF binding site. **Table S7.** GO ID in transcriptome data. **Table S8.** The primers of qRT-PCR.

## Data Availability

All data generated or analysed during this study are included in this published article and its supplementary information files. Phylogeny data are submitted into the Treebase repository (http://purl.org/phylo/treebase/phylows/study/TB2:S28162?x-access-code=e939af3376849ed9741d964ab1b9b1bc&format=html).
