# Supplementary material for: Genome-wide characterization and expression profiling of B3 superfamily during ethylene-induced flowering in pineapple (Ananas comosus L.)
Source: BMC Genomics. 2021 Jul 21;22:561. doi: 10.1186/s12864-021-07854-1 (PMC8296579; doi:10.1186/s12864-021-07854-1)
Supplement: Supplementary file 2 — Additional file 2: Table S1. Physical and chemical properties of B3 genes predictions. Table S2. Ka/Ks calculation of the duplicated pineapple B3 gene pairs. Table S3. One-to-one orthologous relationships between pineapple and Arabidopsis, pineapple and rice. Table S3. One-to-one orthologous relationships between pineapple and Arabidopsis, pineapple and rice. Table S4. Seauence and characteristics of conserved motifs identified in pineapple. Table S5. The cis-element sites of 57 B3 promoters. Table S6. The prediction of ERF binding site. Table S7. GO ID in transcriptome data. Table S8. The primers of qRT-PCR. [file 12864_2021_7854_MOESM2_ESM.docx]

**Genome-wide characterization and expression profiling of B3 superfamily during ethylene-induced flowering in pineapple (*Ananas comosus* L.)**

**Cheng Cheng Ruan^1^, Zhe Chen^1^, Fu Chu Hu^1^, Wei Fan^2^, Xiang He Wang^1^, Li Jun Guo^1^, Hong Yan Fan^1^, Zhi Wen Luo^1^, Zhi Li Zhang^1*^**


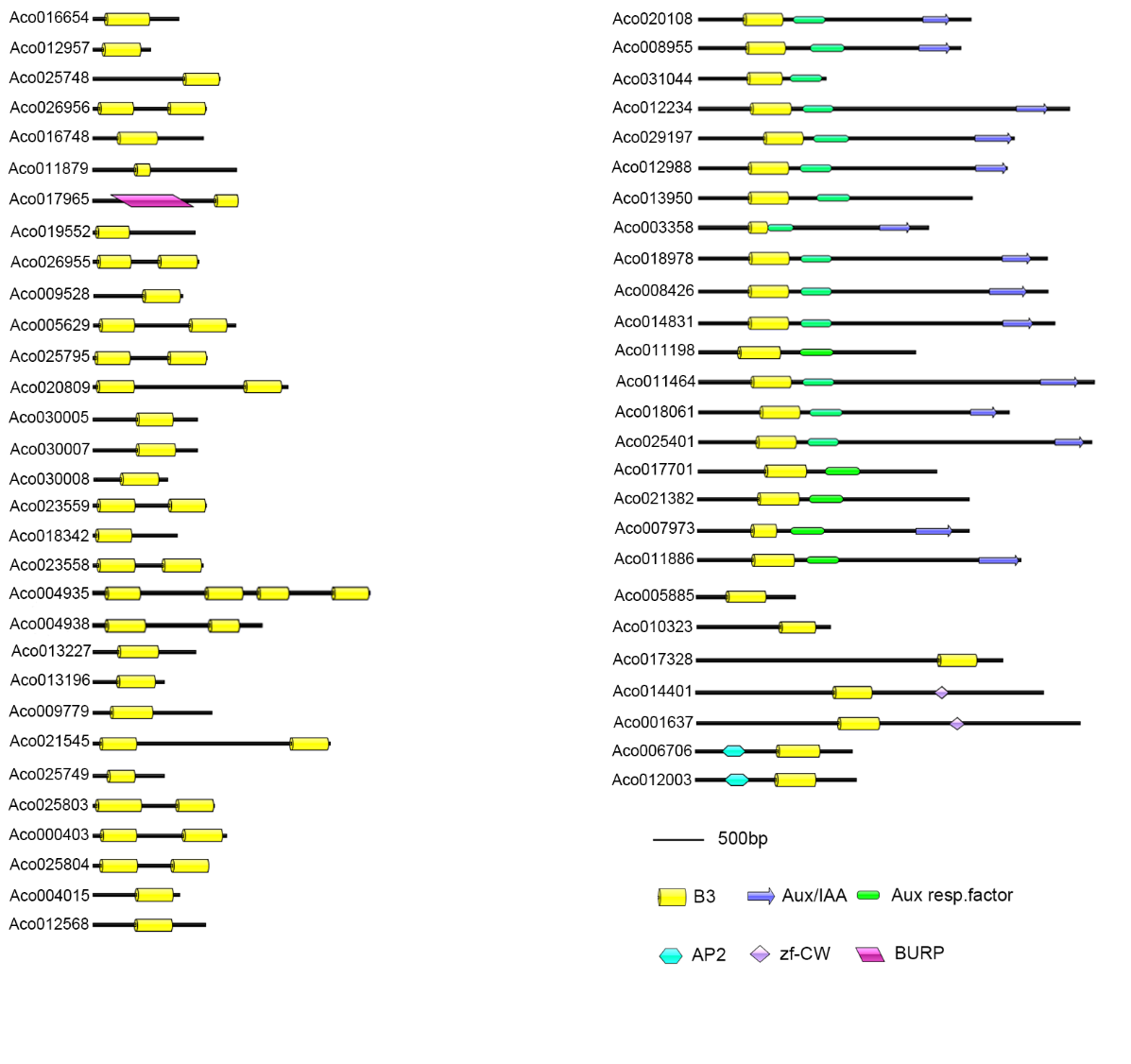


**Fig. S1** Structural domain diagram of B3 genes


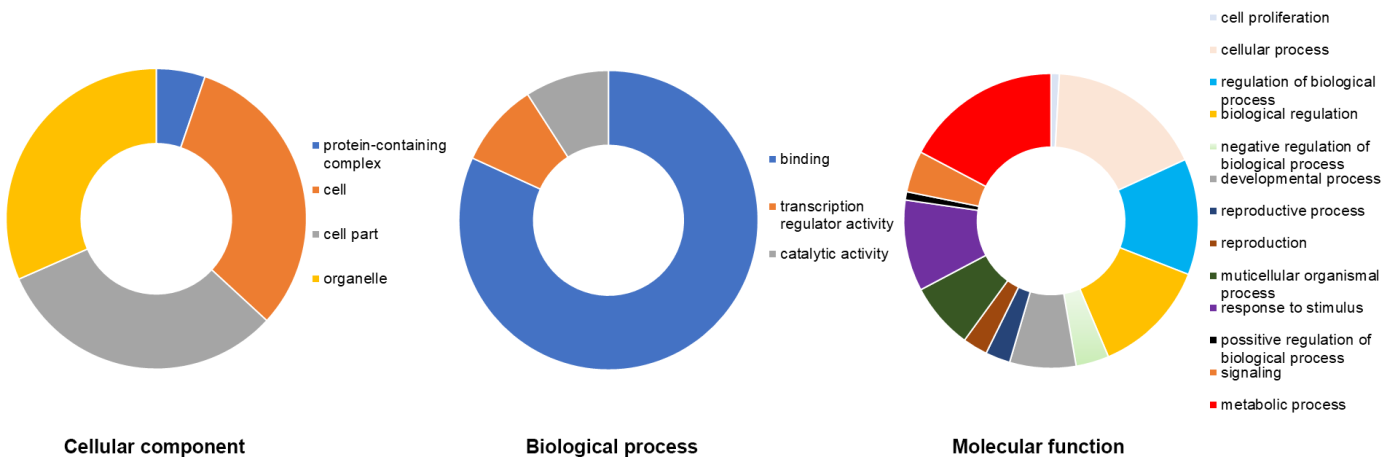


**Fig. S2** Gene ontology analysis of AcB3 proteins. Three categories (cellular component, biological process and molecular function) and terms on level 2 were exhibited by different colors.


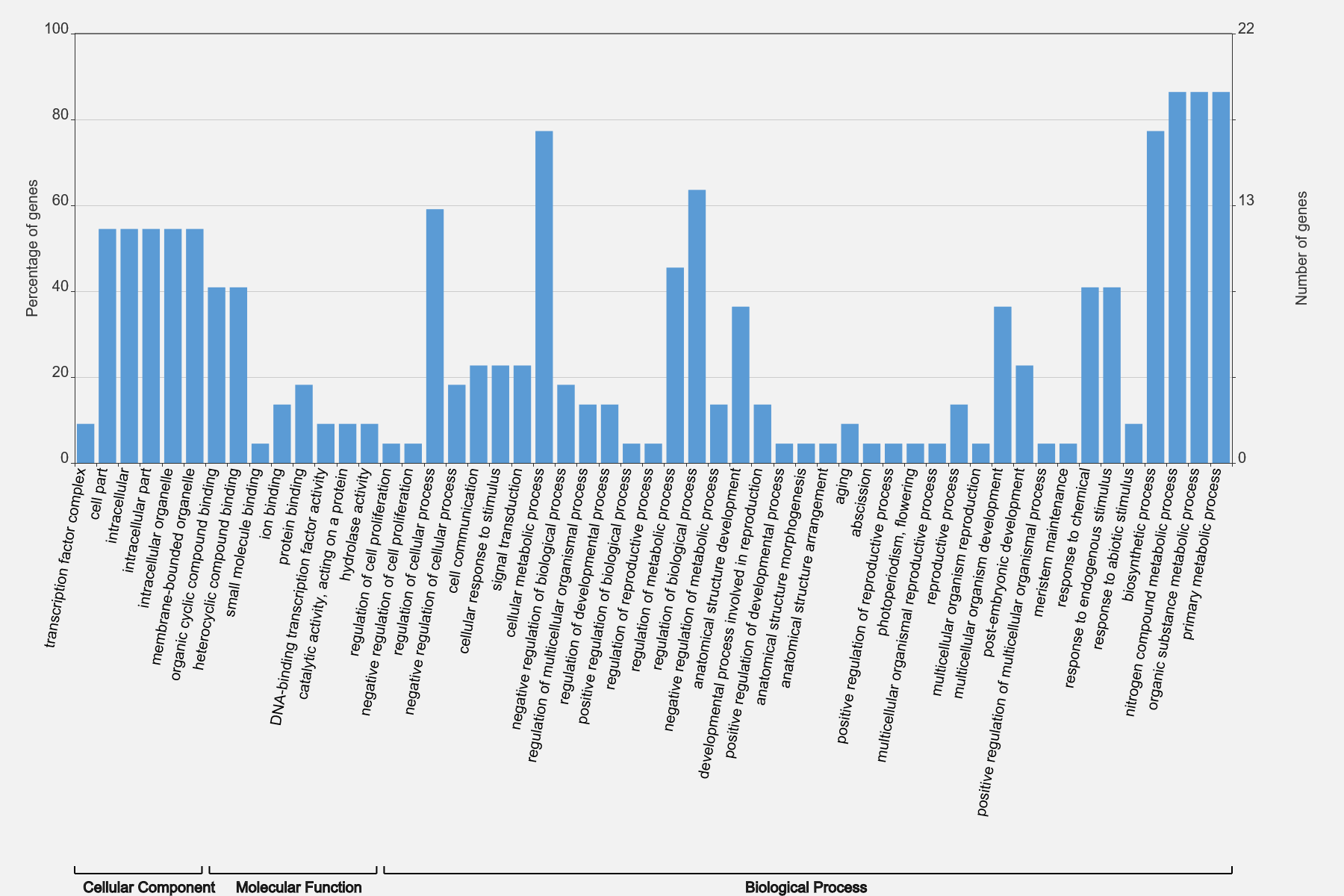


**Fig. S3** GO terms on level 3
